# Evaluation of a simple polytetrafluoroethylene (PTFE)-based membrane for blood-feeding of malaria and dengue fever vectors in the laboratory

**DOI:** 10.1186/s13071-018-2823-7

**Published:** 2018-04-11

**Authors:** Doreen J. Siria, Elis P. A. Batista, Mercy A. Opiyo, Elizangela F. Melo, Robert D. Sumaye, Halfan S. Ngowo, Alvaro E. Eiras, Fredros O. Okumu

**Affiliations:** 1 Environmental Health and Ecological Sciences Department, Ifakara Health Institute, Morogoro, Tanzania; 2 Laboratory of Chemical Ecology of Insect Vectors, Department of Parasitology, Institute of Biological Sciences, Federal University of Minas Gerais, Belo Horizonte, MG Brazil; 3 Barcelona Institute for Global Health (ISGLOBAL), Hospital Clinic, Barcelona, Spain; 4 School of Public Health, Faculty of Health Sciences, University of the Witwatersrand, Park Town, South Africa; 5 Institutes of Biodiversity, Animal Health and Comparative Medicine, University of Glasgow, G12, 8QQ, Glasgow, UK

**Keywords:** Artificial feeding, Blood-feeding success, Membrane feeding, *Aedes aegypti*, *Anopheles arabiensis*, *Anopheles gambiae* (*s.s.*), Fecundity, Survival

## Abstract

**Background:**

Controlled blood-feeding is essential for maintaining laboratory colonies of disease-transmitting mosquitoes and investigating pathogen transmission. We evaluated a low-cost artificial feeding (AF) method, as an alternative to direct human feeding (DHF), commonly used in mosquito laboratories.

**Methods:**

We applied thinly-stretched pieces of polytetrafluoroethylene (PTFE) membranes cut from locally available seal tape (i.e. plumbers tape, commonly used for sealing pipe threads in gasworks or waterworks). Approximately 4 ml of bovine blood was placed on the bottom surfaces of inverted Styrofoam cups and then the PTFE membranes were thinly stretched over the surfaces. The cups were filled with boiled water to keep the blood warm (~37 °C), and held over netting cages containing 3–4 day-old inseminated adults of female *Aedes aegypti*, *Anopheles gambiae* (*s.s.*) or *Anopheles arabiensis*. Blood-feeding success, fecundity and survival of mosquitoes maintained by this system were compared against DHF.

**Results:**

*Aedes aegypti* achieved 100% feeding success on both AF and DHF, and also similar fecundity rates (13.1 ± 1.7 and 12.8 ± 1.0 eggs/mosquito respectively; *P* > 0.05). *An. arabiensis* had slightly lower feeding success on AF (85.83 ± 16.28%) than DHF (98.83 ± 2.29%) though these were not statistically different (*P* > 0.05), and also comparable fecundity between AF (8.82 ± 7.02) and DHF (8.02 ± 5.81). Similarly, for *An. gambiae* (*s.s.*), we observed a marginal difference in feeding success between AF (86.00 ± 10.86%) and DHF (98.92 ± 2.65%), but similar fecundity by either method. Compared to DHF, mosquitoes fed using AF survived a similar number of days [Hazard Ratios (HR) for *Ae. aegypti* = 0.99 (0.75–1.34), *P* > 0.05; *An. arabiensis* = 0.96 (0.75–1.22), *P* > 0.05; and *An. gambiae* (*s.s.*) = 1.03 (0.79–1.35), *P* > 0.05].

**Conclusions:**

Mosquitoes fed *via* this simple AF method had similar feeding success, fecundity and longevity. The method could potentially be used for laboratory colonization of mosquitoes, where DHF is unfeasible. If improved (e.g. minimizing temperature fluctuations), the approach could possibly also support studies where vectors are artificially infected with blood-borne pathogens.

## Background

Laboratory colonization of disease-transmitting mosquitoes is essential for studying the biology, behaviors and physiology of these mosquitoes, as well as their roles in pathogen transmission and how they can be controlled [1, 2]. Blood-feeding of female mosquitoes is a fundamental part of such laboratory colonization efforts, as it is essential for egg development [2]. In earlier studies, human arms and live animals such as guinea pigs, mice, rats, hamsters and chickens have been utilized for feeding mosquitoes in the laboratories [2–6]. Some of the mosquito species, such as *Anopheles gambiae* (*s.s.*) are, however, highly anthropophagic [7, 8], and require human blood meals for a successful colonization [2]. The common use of human arm-feeding, however, causes great distress to the volunteers and is cumbersome, unsuitable for mass-rearing, and raises ethical concerns about possible accidental transmission of diseases. Therefore, a replacement using indirect sources of blood meals for mosquitoes is needed.

Blood-feeding using laboratory animals is a commonly considered as an alternative, and has added advantages, such as the blood being always available alongside natural feeding cues [9]. Feeding on laboratory animals could, however, also result in accidental disease transmission and discomfort to the animals due to hypersensitivity to mosquito bites. Further to this, since mosquitoes readily adapt to different hosts based on availability and abundance [7], it can happen that mosquitoes raised on animal blood might not have similar behavioral responses as those raised on human blood. This would make them less useful for experiments related to human-vector interactions and mosquito host-seeking behaviors. Other disadvantages of using animals to feed mosquitoes include the regulatory concerns raised by animal welfare groups, and expenses related to proper animal housing and maintenance, which can be very expensive and possibly prohibitive for small laboratories [10, 11]. Besides this, the quality of experimental results may be difficult to standardize in cases where the treatment and care of laboratory animals is not quality-assured [12]. Additionally, restraining laboratory animals for blood-feeding can also be inconvenient and difficult to achieve. The above challenges strongly suggest the need for alternatives that do not involve using humans or animals, yet are of low-cost and convenient.

There have been several studies on development and testing of artificial feeding methods. Many of these studies have investigated the potential of these artificial membranes as alternatives for maintaining mosquito colonies in laboratory studies, and possibly for mass-rearing. Indeed, artificial membranes for mosquito blood-feeding have already been used for several decades, mostly for specific experimental studies with blood-sucking insects. In a 1958 review by Tarshis [6], it was noted that membranes were already being used for laboratory blood-feeding of arthropods as early as 1912. Later examples have included formats such as: (i) the early configurations used by L. Owens for membrane feeding of blood to *Culicoides* [13]; (ii) an innovative combination of conical tubes, glycerol and parafilm, originally used by Costa-da-Silva et al. [5] for blood-feeding *Aedes aegypti* mosquitoes; (iii) the use of cattle collagen sausage membranes to facilitate feeding of *Ae. aegypti* mosquitoes [14]; and (iv) a unique design consisting of cover glass, plastic dish covers and flasks [15] for infecting mosquitoes with microfilariae of *Brugia pahangi* worms. Various other membrane based designs, mostly using parafilm, collagen or latex have also been described [16, 17].

Some of these artificial feeding assays have also been tested as a method to infect mosquitoes with disease pathogens, or to determine infection and transmission thresholds of different mosquito borne pathogens including viruses, protozoans like *Plasmodium* spp., and filarial worms in the mosquitoes [18, 19]. One of the earliest reports on using membrane feeders to infect mosquitoes was by Rutledge et al. [20], who used autoclavable heat-resistant glass and stainless steel, making them suitable for conducting infection studies safely and without contamination. Different versions of this original setup are still in use in many laboratories today. Recent reports include Bonnet et al. [21], and Sattabongkot et al. [22], who evaluated artificial systems consisting of glass feeders closed with a Baudruche membranes for *Plasmodium* infection studies. Similar systems had previously been used for laboratory feeding of blackflies (*Similium damnosum*), using both silicon and Baudruche membranes [23], but also for the ixodid ticks, *Ripicephalus appendiculatus*, using Baudruche membranes bearing olfactory and tactile cues to promote attachment and feeding [24]. A common feature of artificial membrane feeders is that they can be used with blood meals from multiple vertebrate sources. Mammalian sources include bovine, chicken and human blood from volunteers or blood banks [25].

A common caveat for membrane feeders is the need to maintain temperatures of the blood and the feeding surfaces at 37 °C, for mosquitoes to feed optimally [26]. This temperature can be maintained by using water baths [14], glycerol or vegetable oils [27], but other systems have more elaborate mechanized systems with circulating baths [20]. For many insect laboratories, a lack of efficient mosquito-feeding methods that are simple to implement and cost-effective remains a major concern, for mass-rearing and also regular laboratory colonization of mosquitoes. There is also a need for simple systems that can be readily adapted for maintaining and studying mosquitoes in the field. Recently, a simple low-cost method for laboratory blood-feeding of *Ae. aegypti* and *An. minimus* mosquitoes has been reported by Finlayson et al. [27]. Heparinized cow blood is used in a Petri-dish covered with thinly stretched parafilm membrane, and pre-heated oil to maintain warm temperatures [27]. The Finlayson design was motivated by the need to create artificial membrane feeding techniques that are neither labor intensive nor expensive, and could therefore be adopted by different laboratories [27].

Here, we evaluated a simple, field-friendly artificial feeding method, which uses disposable Styrofoam cups and membranes made of polytetrafluoroethylene (PTFE) tapes readily available in local stores, and commonly used as a seal tape in plumbing or gas works. We report results of tests where the system was used for blood-feeding of the dengue fever vector, *Ae. aegypti* and two malaria vector species, *An. gambiae* (*s.s.*) and *An. arabiensis* mosquitoes in Tanzania. We compared the performance of this new system relative to standard human volunteer arm-feeding, by assessing the percentage of mosquitoes that successfully blood-fed (feeding success rate), number of eggs laid (fecundity rate), and daily survival rates of the mosquitoes fed by each method.

## Methods

### Mosquito rearing

Mosquitoes were obtained from an insectary maintained inside the Ifakara Health Institute’s mosquito laboratories, the VectorSphere. The larvae are maintained at 26–28 °C temperature, 75-85% relative humidity, and in 12:12 h (light: dark) photoperiod. The egg batches were collected from the colony on filter papers in egg-laying bowls, and were then washed out from the filter paper into plastic basins containing water. After the eggs hatched, larvae were fed daily with TetraMin® ground fish food (Tetra GmbH, Herrenteich Melle, Germany) until pupation. A plastic pipette was used to transfer pupae from the basins into disposable cups, which were then placed inside 30 × 30 × 30 cm cages, where they emerged into adults. Emerged female and male adult mosquitoes were kept together for 3–4 days to allow mating. Adults were provided with 10% sugar solution *via* filter paper, but were starved for 12 h prior to each experiment.

### Apparatus used for artificial feeding (AF) of mosquitoes

**Fig. 1:**
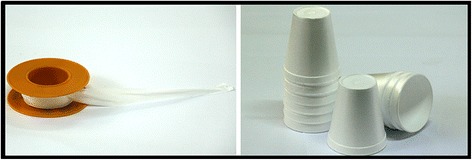
Polytetrafluoroethylene (PTFE) seal tapes and Styrofoam cups, constituting the main components of the apparatus for artificially blood-feeding female adult mosquitoes

**Fig. 2:**
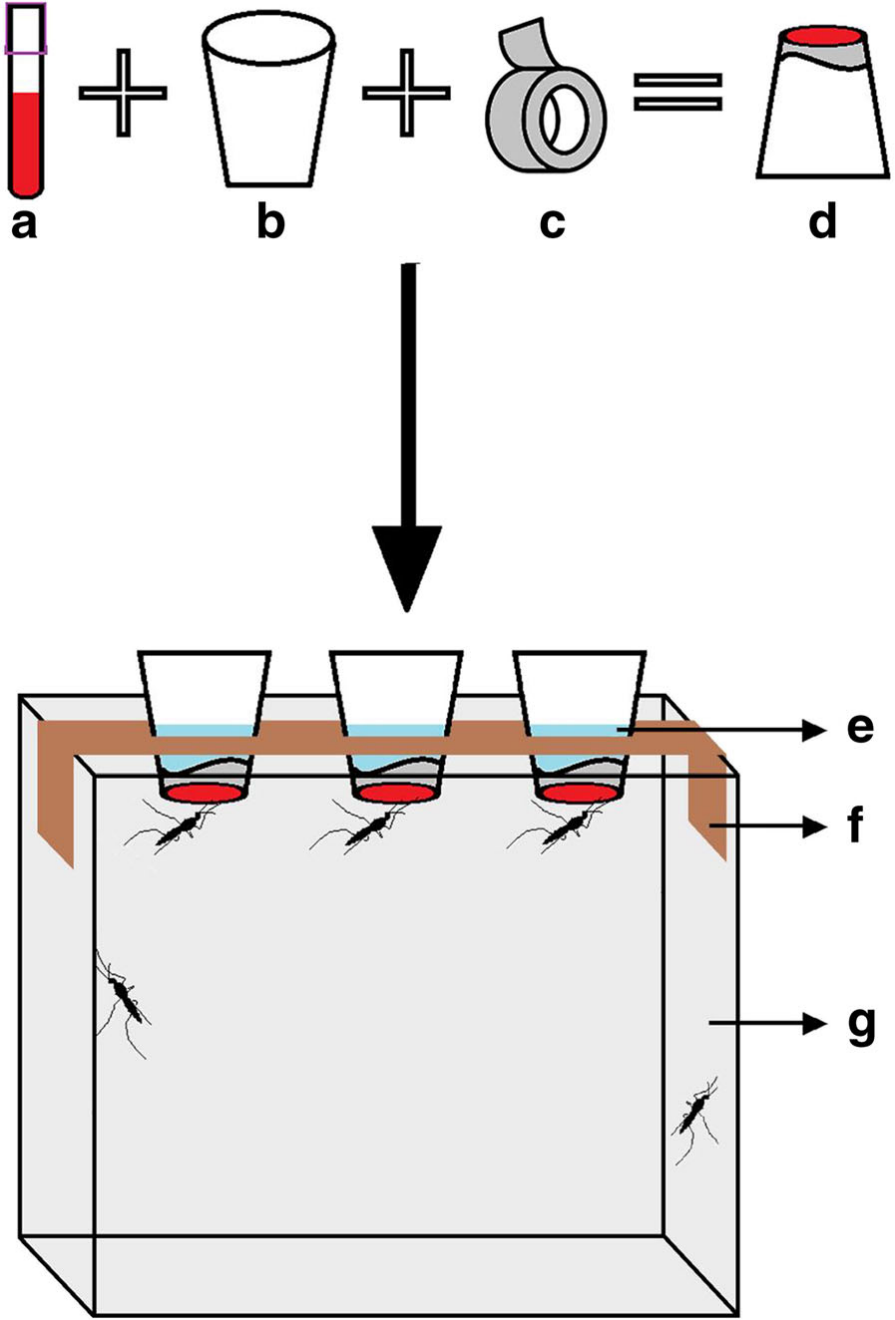
Schematic diagram of the polytetrafluoroethylene (PTFE) membrane-based blood-feeding system. **a** EDTA tube with blood. **b** 180 ml Styrofoam cup. **c** PTFE roll. **d** Prepared PTFE membrane-based blood meal on the underside of the Styrofoam cup. **e** Warm water necessary to achieve 37 °C blood meal. **f** Wooden holding board for holding the inverted cups with PTFE-covered blood meal. **g** Mosquito cage measuring 30 × 30 × 30 cm

**Fig. 3:**
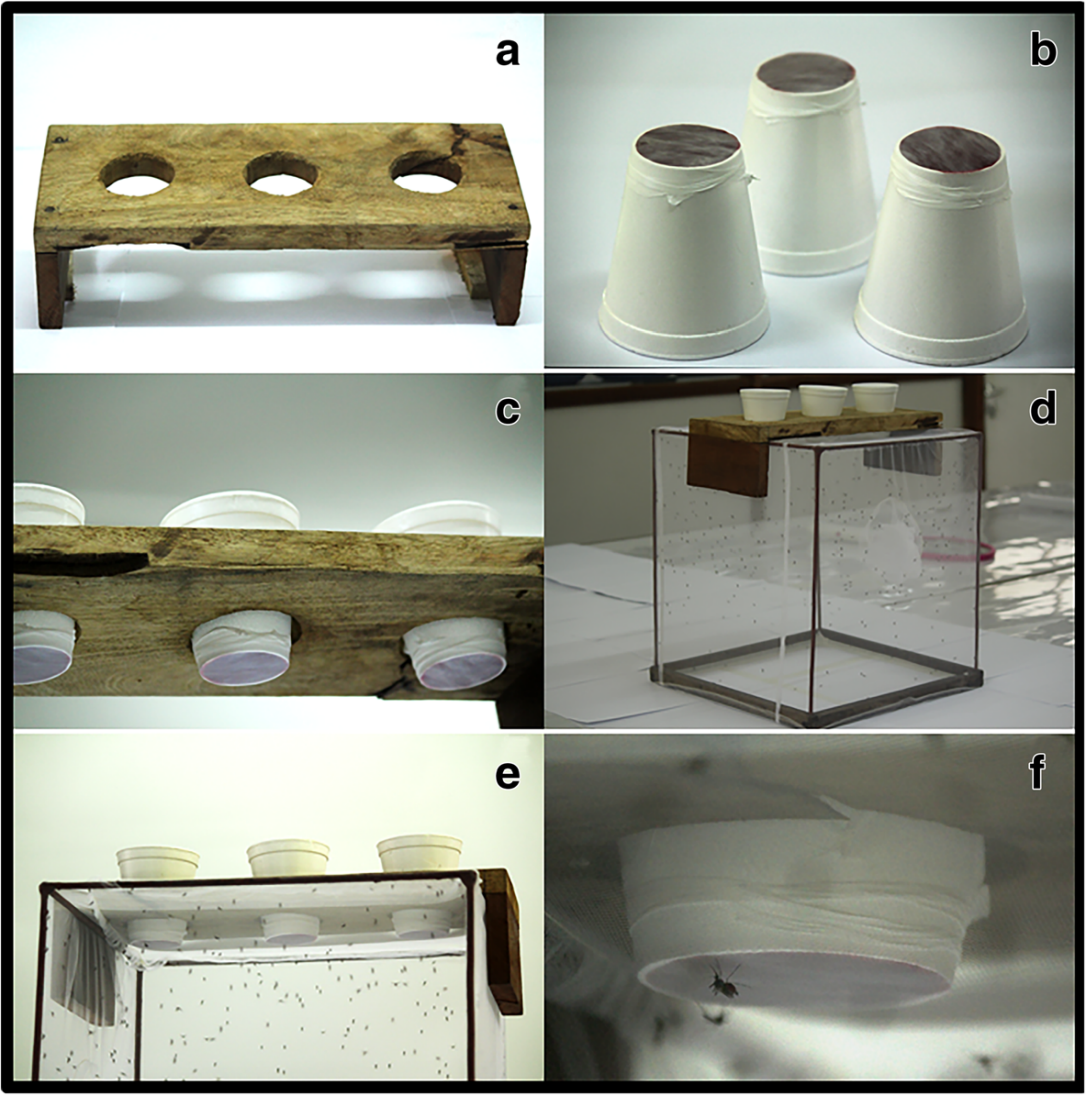
Polytetrafluoroethylene (PTFE) membrane-based blood-feeding system. **a** Wooden holding board. **b** Inverted Styrofoam cups holding ~ 4 ml of bovine blood on the underside. **c** Styrofoam cups containing warm water to ensure the blood-meal is at ~ 37 °C temperature, and held in place using the wooden holding board. **d** Wooden board placed on the top of the cage containing mosquitoes. **e**, **f** Mosquitoes feeding through the membrane

The artificial feeding (AF) apparatus was designed and made on site with simple, low-cost and easily accessible materials, all obtained locally. The main items were disposable Styrofoam cups (180 ml size), which were inverted to hold blood on the underside, and a piece of polytetrafluoroethylene (PTFE) seal tape (~ 80 × 20 × 0.2 mm); only one piece of PTFE membrane was stretched thinly to form the necessary membrane to hold the blood on the underside of the Styrofoam cups (Fig. 1). A schematic illustration of how the membrane and the Styrofoam cups were used is provided in Fig. 2. We also constructed and used a wooden holding board with three round holes to insert the cups (Fig. 3a, c). The holding board was placed on top of the mosquito cages to support the cups during the artificial feeding experiments (Fig. 3d, e).

### Artificial membrane feeding (AF) of mosquitoes

To demonstrate the functionality of this system, bovine blood was used for the artificial feeding. The blood was collected by a veterinarian (RDS) and placed into 10 ml vacutainer tubes (Becton & Dickinson Vacutainer^TM^ (Franklin Lakes NJ, USA) with the anticoagulant, ethylenediaminetetraacetic acid (EDTA). The Styrofoam cups were inverted and 4 ml of bovine blood placed at the bottom surface of the cups. One small piece of the PTFE membrane (~ 80 × 20 × 0.2 mm) was then thinly stretched over the blood drop, for the mosquitoes to imbibe blood easily. To mimic the temperature of vertebrate blood, hot water was added in the cups to keep the blood warm, at about 37°C. This temperature was maintained throughout the blood-feeding process by checking water temperature by using a thermometer, pipetting out the water and adding warm water, without disturbing the feeding process. To simplify the procedures during these studies, this water-replacement approach was used instead of an automated water bath which could, however, be readily added onto our system. To start the feeding process, the wooden holding board was placed on top of the mosquito cages, then the Styrofoam cups inserted as shown in Fig. 3c. Each of the cages contained 200 of 3–4 days-old inseminated female mosquitoes that had been starved for 12 h prior to blood-feeding. Each feeding process lasted for 20 min. All the males were excluded prior to experimentation.

### Direct human feeding (DHF) of mosquitoes

Direct host feeding was done by a consenting adult male human volunteer, by inserting his arm into the mosquito cages, as was the standard practice for maintaining insectary colonies. Only two hundred mated female mosquitoes were kept in the cages (30 × 30 × 30 cm), and were allowed to feed directly for 20 min in each experiment. The volunteer wore gloves to protect the wrist and fingers, during the feeding.

### Assessing blood-feeding success of mosquitoes fed by artificial or direct arm-feeding

Two hundred laboratory reared female mosquitoes per species were introduced into each two mosquito feeding cages. In one feeding cage, the AF apparatus was placed on top while in the other cage (Fig. 3) the human volunteer exposed his arm. Mosquitoes were left to feed for 20 min, after which the blood-fed females were visually identified and aspirated from the cages and counted. Three experimental replicates were conducted, each with 200 female mosquitoes for each species.

### Assessing fecundity rates in mosquitoes fed by artificial (AF) or direct human feeding (DHF)

All engorged females were kept in separate cages labeled by species and feeding method. They were maintained on 10% sugar solution at the same temperature and humidity conditions as the main insectary. Three days post-feeding, small oviposition cups containing wet cotton wool and filter paper (12 cm in diameter), were placed in each cage for the females to lay eggs. The mosquitoes were allowed to lay eggs for two days, but the egg cups were retrieved daily. The eggs laid each day were counted under a stereo microscope and recorded. The mosquito species, feeding method and the number of live mosquitoes in each cage was also recorded.

### Assessing survival rates of mosquitoes fed by artificial or direct arm-feeding

The survival rate was monitored for all the mosquito species, i.e. *Ae. aegypti*, *An. gambiae* (*s.s.*) and *An. arabiensis*, from the initial blood meal until they died. This was done by transferring the mosquitoes to respective clean cages, where they continued to be fed on blood and 10% sugar solution. Mortality was recorded every 24 h in each cage until all the mosquitoes died.

### Data analysis

Blood-feeding success was calculated as the number of mosquitoes that were fully or partly-fed as a fraction of the total number of mosquitoes exposed:

$$ \mathrm{Feeding}\ \mathrm{success}\ \mathrm{rate}\ \left(\%\right)=\left(\mathrm{number}\ \mathrm{of}\ \mathrm{females}\ \mathrm{engorged}\ \mathrm{after}\ 20\ \min\ \mathrm{feeding}/\mathrm{total}\ \mathrm{number}\ \mathrm{of}\ \mathrm{females}\ \mathrm{in}\ \mathrm{the}\ \mathrm{feeding}\ \mathrm{cage}\right)\times 100 $$

Engorged mosquitoes were identified by visual observation of a swollen, red-colored abdomen. With regards to fecundity, the number of eggs laid by the mosquitoes was counted under a stereo microscope, assuming that all the blood-fed mosquitoes in the cage had an equal opportunity to lay eggs. Fecundity rates were calculated by dividing total number of eggs laid in each cage by total number of mosquitoes present in the cage.

Further analysis was performed in the open source software, R v.3.3.2 [28], as follows: a paired t-test was used to analyze the feeding rate and fecundity rate of the mosquitoes, with statistically significant differences determined at *P* = 0.05. The survival rate was calculated as the percentage of female mosquitoes that survived each subsequent day after the first blood meal. Cox proportional hazard model was used to analyze daily survival data, and to estimate the daily probabilities of mosquitoes after feeding on either AF or DHF. Probabilities of survival in the form of hazard ration (HR) were generated by comparing the survival curves of mosquitoes fed by AF and DHF methods, with DHF as reference. In these tests, HR values of 1.0 indicated equal mortality rates between the two feeding methods, HR < 1 indicated lower mortality rates by AF compared to DHF, and HR > 1 indicated higher mortality rates by AF compared to DHF.

## Results

### Blood-feeding success

**Fig. 4:**
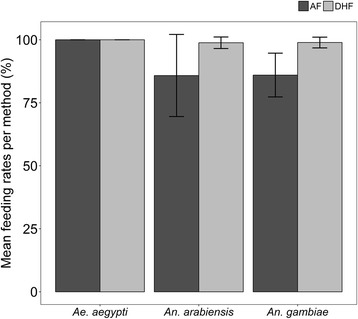
Average feeding (mean ± SD) success rates by *Aedes aegypti*, *Anopheles arabiensis* and *Anopheles gambiae* (*s.s.*) given 20 min exposure to blood meals *via* the PTFE membrane (AF) and direct human feeding (DHF) methods. Error bars represent standard errors with 95% CI

All mosquito species tested, i.e. *Ae. aegypti*, *An. gambiae* (*s.s.*) and *An. arabiensis* achieved 85% or higher feeding success rates by both AF and DHF methods. *Aedes aegypti* had the best feeding outcomes, achieving 100% success rates in both feeding methods (Table 1, Fig. 4). On the other hand, the average feeding success rate of *An. arabiensis* that fed artificially was 85.83 ± 16.28, while for those that fed directly on human arm reached 98.83 ± 2.29 feeding success (Table 1, Fig. 4). Lastly, the average feeding success rate for *An. gambiae* (*s.s.*) mosquitoes fed by DHF was 98.92 ± 2.65, higher than those fed by AF, which was nonetheless also high at 86.00 ± 10.86, as shown in Table 1 and Fig. 4.

**Table 1:** Blood-feeding rates of mosquitoes of different species fed *via* either the PTFE-membrane based artificial feeding method (AF) or the direct host feeding method (DHF)

Mosquito species	Artificial feeding (AF) (%)	Direct human feeding (DHF) (%)	Paired *t*-test
	Mean ± SD	Mean ± SD	
*Anopheles arabiensis*	85.83 ± 16.28	98.83 ± 2.29	*t*_(5)_ = -1.7372, *P* = 0.142
*Anopheles gambiae*	98.92 ± 2.65	86.00 ± 10.86	*t*_(5)_ = 3.4637, *P* = 0.018
*Aedes aegypti*	100	100	na

*Abbreviation*: na, not available

### Fecundity rates

**Fig. 5:**
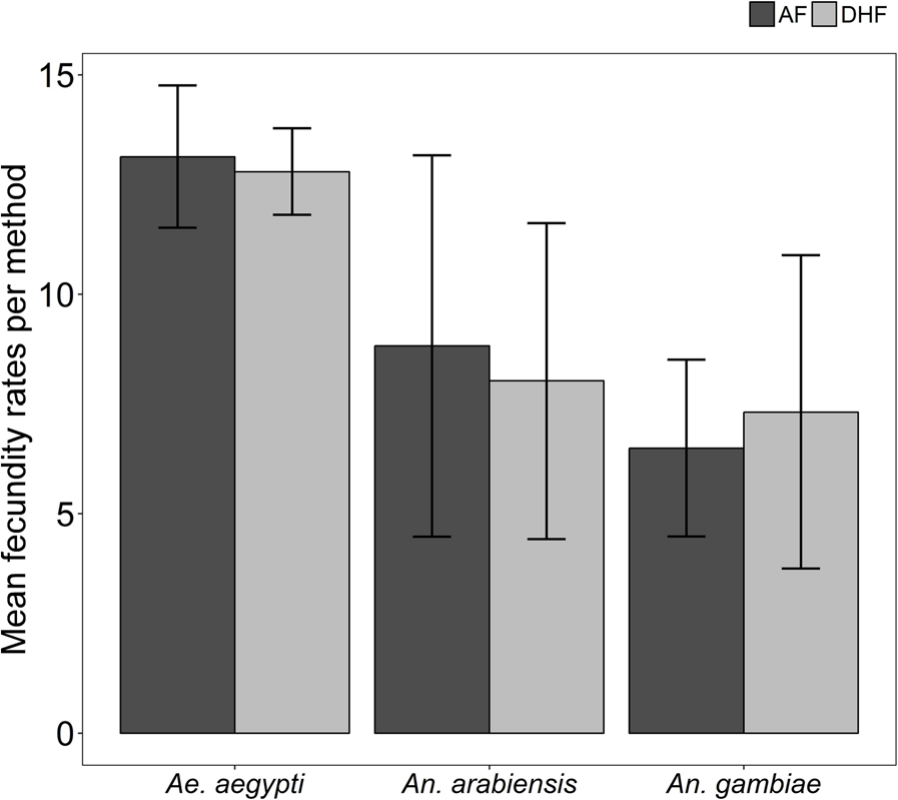
Mean number (mean ± SD) of eggs laid by individual *Aedes aegypti*, *Anopheles arabiensis* and *Anopheles gambiae* (*s.s.*) mosquitoes fed using AF and DHF methods. The error bars represent standard errors with 95% CI

Generally, fecundity rates were not affected by the blood-feeding method, in any of the three species tested (Fig. 5). There was a higher survival rate of engorged females observed between the blood-feeding and oviposition time, for all mosquito species with both AF and DHF methods. The mean number of eggs laid per mosquito species was not significantly different from blood-fed by AF and DHF, respectively, for *Ae. aegypti* (13.14 ± 1.65; 12.80 ± 1.00), *An. arabiensis* (8.82 ± 7.02; 8.02 ± 5.81) and *An. gambiae* (*s.s.*) (6.49 ± 2.90; 7.32 ± 5.15) (Table 2, Fig. 5).

**Table 2:** Fecundity rates of mosquitoes of different species fed *via* either the PTFE-membrane based artificial feeding method (AF) or the direct host feeding method (DHF)

Mosquito species	Artificial feeding (AF) (No. eggs/mosquito)	Direct human feeding (DHF) (No. eggs/mosquito)	Paired *t*-test
	Mean ± SD	Mean ± SD	
*Anopheles arabiensis*	8.82 ± 7.02	8.02 ± 5.81	*t*_(9)_ = 1.1664, *P* = 0.273
*Anopheles gambiae*	6.49 ± 2.90	7.32 ± 5.15	*t*_(7)_ = 0.4959, *P* = 0.635
*Aedes aegypti*	13.14 ± 1.65	12.80 ± 1.00	*t*_(3)_ = 1.0091, *P* = 0.387

### Survival rates

**Fig. 6:**
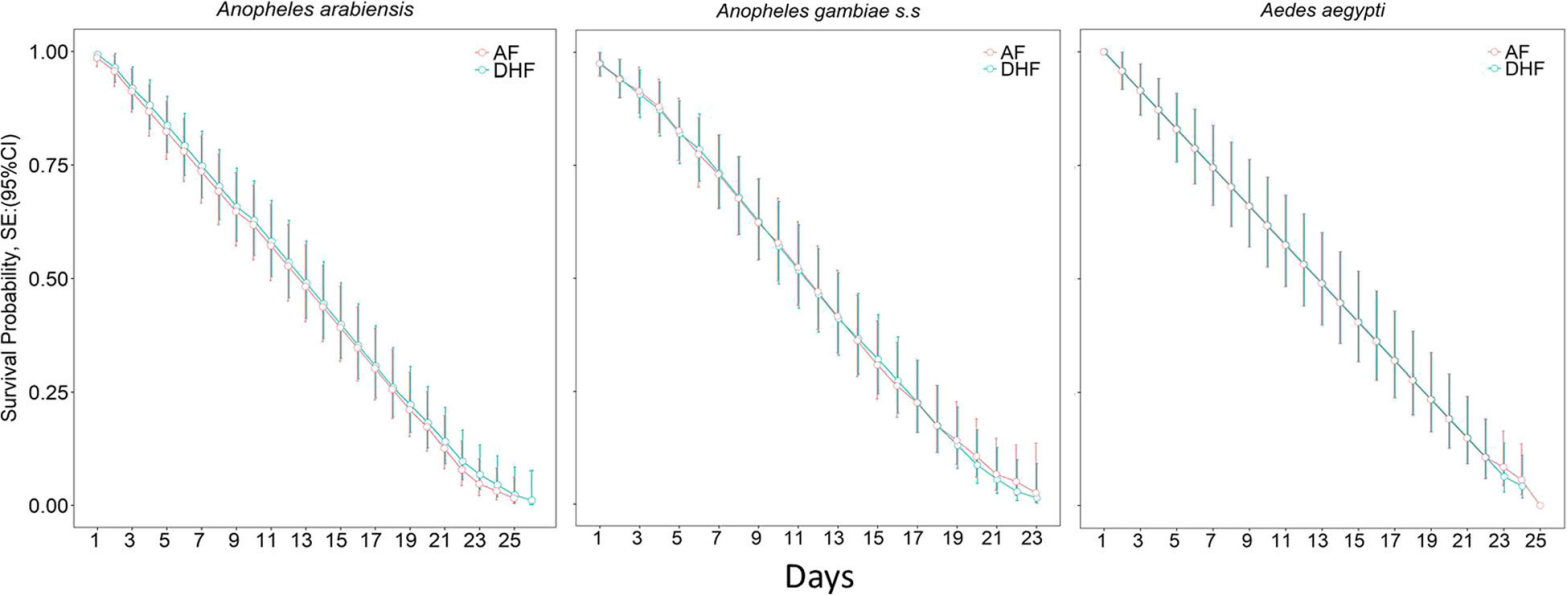
Survival rates of malaria vectors of *Anopheles arabiensis* and Anopheles *gambiae* (*s.s.*), and the dengue fever vector *Aedes aegypti* when blood-fed either by using the PTFE membrane (AF) or direct human arm-feeding. The survival rates were estimated from a Cox proportion hazard regression model with their respective 95% CI

We observed no difference in daily survival rates when mosquitoes were fed on either AF or DHF methods for *An. arabiensis* (Fig. 6). The average daily probability of survival for *An. arabiensis* was only marginally lower when feeding on DHF, compared to AF [Hazard Ratio = 0.957 (0.749–1.222), *P* > 0.05]. Similarly, for *An. gambiae* (*s.s.*) and *Ae. aegypti* there were no statistically significant differences in survival rate found between mosquitoes feeding on either AF or DHF methods [HR = 1.032 (0.787–1.353), *P* > 0.05 and HR = 0.999 (0.746–1.6339), *P* > 0.05], respectively.

## Discussion

Female mosquitoes require blood meals, so they can digest the erythrocytes and plasma proteins to obtain amino acids for synthesizing yolk proteins for their egg production [29]. There have been different ways of feeding mosquitoes in the laboratory, with most relying on direct feeding on live animals such as guinea pigs, hamsters and mice, as well as humans. However, these methods have some limitations, such as poor standardization, ethical concerns, human volunteer safety concerns, animal welfare concerns and high costs. The findings from this study have demonstrated that a simple method for artificially blood-feeding mosquitoes can be created using a novel polytetrafluoroethylene (PTFE) material as the main feeding membrane. The membrane can be fitted with locally available materials such as disposable Styrofoam cups. This system could potentially be used to maintain malaria and dengue fever vectors in the laboratory, but more importantly it is simple, affordable, efficient and suitable for multiple purposes. The materials are locally available, and the PTFE membrane itself is commonly used as plumber’s tape even in peripheral towns.

We assessed the system and demonstrated its potential on the basis of three important parameters, i.e. (i) whether the mosquitoes exposed to the feeding system would successfully blood feed, compared to those offered a regular human host blood-meal; (ii) whether the blood-fed mosquitoes could lay as many eggs as those fed on regular human host blood; and (iii) whether the mosquitoes blood-fed using the new system would survive as long as those given regular host blood meal.

Our results showed that the feeding success rate by all mosquitoes tested was similar in both the AF and DHF method, despite the known differences in blood-feeding behaviors exhibited by the mosquitoes species studied [7]. For example, *An. gambiae* (*s.s.*) is a highly anthropophilic mosquito [7], while its sibling species, *An. arabiensis,* often has multiple blood-hosts, depending on availability of the hosts [30]. However, the findings that direct blood-feeding with human volunteer arms was marginally better, indicates the general preferences of all the mosquitoes tested to human rather than other host blood. However, this assay was not designed to directly compare these host differences, thus no direct conclusion can be made here, indeed our study also demonstrated sufficient feeding success rates on both AF and DHF methods.

Blood-feeding success in mosquitoes can be influenced by complete human body emanations including body odor, heat and moisture [30, 31]. Temperatures influence the ability of mosquitoes to successfully blood-feed. Mosquitoes will often suck blood at much lower rates when presented with the blood meal at lower temperatures than that at higher temperature [32]. In the experiments reported by Lusiyana et al. [32] in 2013, the authors concluded that heating increased blood-feeding success in *Ae. aegypti* mosquitoes, but also that anticoagulants such as K_3_EDTA, heparin and sodium citrate could be used to improve feeding rates, fecundity and hatchability of the mosquito eggs, along with the aquatic development of the mosquitoes [32]. For this reason, temperature control is an important aspect of artificial membrane feeding methods, the main aim being to mimic as closely as possible the actual host temperatures. Based on the feeding success findings displayed, the AF method can be used as a way to feed these mosquitoes in the laboratory, but improvements need to be made on the actual apparatus, so that the temperatures can be maintained for longer. Alternative options include the use of heated oil that would retain heat for longer, or an insulation system on the cups, so that the heat is not lost rapidly. Since the feeding process lasts only up to 20 min, a good insulation system that can retain the necessary heat levels, for just that long would be sufficient.

Other than blood-feeding success, many studies have also assessed the variance of fecundity of mosquitoes after feeding on different hosts. In our study, we observed that fecundity rates of the three mosquito species tested were not affected by the feeding methods, which is a crucial outcome. It also proves that each method can be used to maintain mosquito colonies in the insectary. Nonetheless, there may be smaller variations of the mosquito fecundity rates between the two methods tested; for example on *An. gambiae* (*s.s.*) membrane feeding showed a marginal but consistent reduction in fecundity when compared to direct feeding. Deng et al. [33] reported the fecundity differences in membrane feeding method may be due to mosquitoes imbibing serum only at the later part of the feeding process, as separation of the serum and blood cells a few minutes after AF blood-fed method exposure. Additionally, other investigations have found this variation to be caused by different levels in the host blood stream of amino acids necessary for egg production or those that limit egg production [34]. Phasomkusolsil et al. [35] on the other hand, reported that mosquito body size, species and size of the blood meal affect mosquito fecundity. Therefore, findings from our study demonstrate that the fecundity rate of mosquitoes does not appear to be affected significantly by the blood source for the species studied. However, we also observed that the egg counts reported here are far lower than the expected numbers in both human-fed and PTFE-fed mosquitoes. We believe this difference is because the data presented are summaries in cages where there may have been mosquitoes with very low egg counts. Besides, we considered only the first egg batches to ensure standardization, and ignored subsequent batches as mosquitoes were not blood-fed after the initial egg-laying. This means any increases in egg counts usually experienced in the second and third egg batches may not have been observed in this experiment.

Lastly, mosquitoes fed using the new apparatus here did not have different daily survival probabilities than mosquitoes of same species fed using the direct human arms. The exceptions were in *An. arabiensis*, where survival rates were marginally lower among those that had blood-fed *via* AF, compared to those that had blood-fed *via* DHF. While in *An. gambiae* (s.s.) blood-feeding success was higher in DHF than in AF, possibly indicating the strong anthropophilic tendencies of this vector. Perhaps it was missing a phagostimulant in the membrane, such as rubbing the human skin in the PTFE prior to mosquito feeding.

One minor limitation of this work was that we used only bovine blood for the artificial blood-feeding and only human blood for the direct arm-feeding. For this reason, any slight differences that observed could be associated with differences in blood type, rather than differences in feeding methods. However, in all cases we observed similarities in feeding success, fecundity and daily survival rates, or only marginal differences. It can be argued that the benefits of using the PTFE membrane based apparatus may be sufficient to conceal any differences related to blood type. Moreover, in the course of improving this system, it will be important to conduct studies where the blood type is the same in the different blood-feeding systems.

A second limitation was that we assessed fecundity of the mosquitoes in pools rather than for individual mosquitoes. We hypothesized that excessive stresses resulting from the shifting of mosquitoes between cages, would affect egg-laying. We therefore opted to only move them from the mating cages to the feeding cages where they were also provided with oviposition basins, and did not evaluate mosquito fecundity at individual level. We understand, however, that this pooled assessment may misrepresent data from mosquitoes that either lay too many eggs or too few eggs. Nonetheless, we have included variation data in our data summaries, by way of confidence intervals. This data therefore offers sufficient information in the parameters of interest between AF and DHF, though we also recommend that future studies should consider assessment of the number of counts of eggs laid per mosquito.

In summary, the feeding rate, fecundity rate and survival rate of *Ae. aegypti*, *An. gambiae* (*s.s.*) and *An. arabiensis* females were not significantly affected by the feeding method. On the contrary, the PTFE membrane blood-feeding system was found to be a potentially desirable alternative to feed mosquitoes in the insectary. Using this specific demonstration as a platform, our method could be further developed to create a system that is advantageous for both experimental and rearing purposes.

## Conclusions

Blood-feeding success, fecundity and survival rates for *Ae. aegypti* mosquitoes were generally similar between AF and DHF. For *An. arabiensis* and *An. gambiae* (*s.s.*) there were marginal but non-significant differences in these parameters between the methods. The novel AF method, which uses locally-available PTFE membranes and disposable cups, could potentially be used to support laboratory colonization and mass-rearing of mosquito vectors, especially where DHF is not feasible. With additional improvements, e.g. adding features to minimize temperature fluctuations, the approach could possibly also support transmission studies where mosquitoes are artificially infected with blood-borne pathogens. Further assessments are therefore necessary to improve the method and determine its suitability for studies where mosquitoes are artificially infected with blood-borne pathogens, such as *Plasmodium* spp.

## Abbreviations

AF: Artificial feeding
DHF: Direct human feeding
EDTA: Ethylenediaminetetraacetic acid
IHI: Ifakara Health Institute
MRDT: Malaria rapid diagnostic test
NIMR: National Institute for Medical Research, Tanzania
PTFE: Polytetrafluoroethylene
